# Cortical excitability in human somatosensory and visual cortex: implications for plasticity and learning – a minireview

**DOI:** 10.3389/fnhum.2023.1235487

**Published:** 2023-08-17

**Authors:** Hubert R. Dinse, Oliver Höffken, Martin Tegenthoff

**Affiliations:** Department of Neurology, BG Universitätsklinikum Bergmannsheil, Ruhr University Bochum, Bochum, Germany

**Keywords:** paired-pulse behavior, cortical inhibition, plasticity, perceptual learning, GABA

## Abstract

The balance of excitation and inhibition plays a key role in plasticity and learning. A frequently used, reliable approach to assess intracortical inhibition relies on measuring paired-pulse behavior. Moreover, recent developments of magnetic resonance spectroscopy allows measuring GABA and glutamate concentrations. We give an overview about approaches employed to obtain information about excitatory states in human participants and discuss their putative relation. We summarize paired-pulse techniques and basic findings characterizing paired-pulse suppression in somatosensory (SI) and (VI) visual areas. Paired-pulse suppression describes the effect of paired sensory stimulation at short interstimulus intervals where the cortical response to the second stimulus is significantly suppressed. Simultaneous assessments of paired-pulse suppression in SI and VI indicated that cortical excitability is not a global phenomenon, but instead reflects the properties of local sensory processing. We review studies using non-invasive brain stimulation and perceptual learning experiments that assessed both perceptual changes and accompanying changes of cortical excitability in parallel. Independent of the nature of the excitation/inhibition marker used these data imply a close relationship between altered excitability and altered performance. These results suggest a framework where increased or decreased excitability is linked with improved or impaired perceptual performance. Recent findings have expanded the potential role of cortical excitability by demonstrating that inhibition markers such as GABA concentrations, paired-pulse suppression or alpha power predict to a substantial degree subsequent perceptual learning outcome. This opens the door for a targeted intervention where subsequent plasticity and learning processes are enhanced by altering prior baseline states of excitability.

## Introduction

When the basics of adult cortical plasticity had been established in the early eighties of the last century, two basic types of explanation had been proposed. Besides the idea that new connections are created by growth of axons and/or dendrites, it was suggested that changes of inhibition and excitation result in reorganization of previously ineffective connections which in turn activates neurons previously inactive (Kaas et al., 1983). This has been substantiated by numerous studies showing that GABA (γ-aminobutyric acid) is a major player modulating and shaping neural excitation. Most importantly, during plasticity and learning, GABAergic mechanisms are crucial for maintaining a precise balance between excitation and inhibition (Abbott and Nelson, 2000; Turrigiano and Nelson, 2000; Feldman, 2009; Carcea and Froemke, 2013; Barron, 2021).

## Measuring paired-pulse suppression

To measure signatures of excitation and inhibition in human subjects, the assessment of paired-pulse behavior has become a standard procedure. It consists of application of pairs of stimuli in close succession (paired-pulse stimulation), which can be used as a marker of intracortical excitability in sensory cortices. This approach is somewhat equivalent to paired-pulse transcranial magnetic stimulation (TMS), which is widely used to assess plastic changes in human motor cortex (Kujirai et al., 1993; Reis et al., 2008).

**FIGURE 1:**
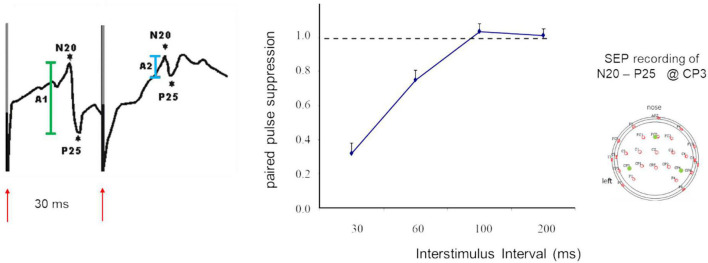
SEP recordings made in SI following paired-median nerve stimulation at an ISI of 30 ms **(left)**. Shown are the N20 P25 components (first response in green, second response in light blue), red arrows indicate timing of stimulation and stimulus artifacts. Paired-pulse suppression as a function of ISI **(middle)**. Recording sites according to the 10-20 system **(right)**. Modified according to Höffken et al. (2007).

When applied in sensory areas, paired-pulse suppression describes the outcome of stimulation with very short interstimulus intervals (ISI) that the cortical responses to the second stimulus are significantly suppressed compared to the first stimulus. Paired-pulse suppression is quantified by calculating the ratio of the amplitude of the second response divided by the amplitude of the first response. Small amplitude ratios indicate strong paired-pulse suppression, while large ratios indicate little paired-pulse suppression which is taken as a marker for enhanced excitation. In studies of the paired-pulse behavior in the hand and finger representations of somatosensory primary cortex (SI), often electrical stimulation of the median nerve is employed (Figure 1). Typically, ISIs in the range of 30 ms produce reliable paired pulse suppression (Allison, 1962; Höffken et al., 2007, 2010, 2013a; Lenz et al., 2012).

To measure paired-pulse behavior in analogy to investigations in the visual cortex (VI) we applied a recently established approach consisting of recording VEPs following patterned paired visual stimulation (Höffken et al., 2008, 2009). Due to the different sensory pathways in touch and vision, and due to different types of subcortical processing, response latencies differ markedly in both modalities, which affects paired-pulse behavior. Therefore, in VI, significant suppression can be recorded at ISIs in the range of 100 ms (Höffken et al., 2008, 2009). However, some differences in methodology should be emphasized. In contrast to studies in SI, which use electrical stimulation of the median nerve and recordings of the N20/P25 component in the SEPs, VI studies employ visual pattern stimulation with recordings of the P100 VEP component. The resulting broad VEP responses make it impossible to use shorter ISIs than around 80 ms, as the response peaks tend to melt into each other at shorter ISIs. It is therefore conceivable that the paired-pulse behavior is more similar in both systems. This possibility is supported by the fact that the suppression ratio in SI at ISIs of 30 ms is in the range of <0.4 (Höffken et al., 2007), while suppression ratios in VI measured at ISIs of 80 ms are in the range of >0.6 (Höffken et al., 2008); cf. chapter “Paired-pulse behavior as an inhibition marker” and “Inhibition markers as predictors of subsequent performance changes.”

## Reflect measurements of cortical excitability of somatosensory and visual cortex a global state?

While there is a clear role of cortical excitability in plasticity and learning, little emphasis has been put on the aspect of how global an individually assessed level of excitability is. Is it limited to within a small cortical representational zone, is it the same across hemispheres and across different sensory modalities? Individual paired-pulse suppression obtained from recordings made within a few minutes after each other in left somatosensory (SI) and primary visual cortex (VI) were comparable although differing in absolute values (Schloemer et al., 2020). However, according to a linear correlation analysis, the amount of paired-pulse suppression measured in SI and VI was unrelated (*p* = 0.951, *r* = −0.012). According to these data, in SI and VI high and low levels of excitability can co-exist in individual participants. The data imply that paired-pulse behavior reflect the properties of sensory processing in local patches of cortex. This high locality might have important implications for crossmodal learning experiments. More important, these data support the view that cortical excitability measured by paired-pulse techniques can be used as a reliable marker characterizing the currently prevailing excitatory state within a circumscribed patch of cortex (Schloemer et al., 2020).

## Paired-pulse behavior as an inhibition marker

Paired-pulse behavior is a reliable marker for obtaining information about the inhibition-excitation status in cortical areas. Therefore, measuring paired-pulse behavior is widely used in animal models, and in particular in human studies because of the non-invasive nature. However, the mechanisms that underlie paired-pulse behavior are not fully understood. Often, the phenomenon of paired-pulse suppression has been subsumed as short-term plasticity (Zucker and Regehr, 2002). Whole-cell recordings made in rat auditory cortex demonstrated that at short ISIs GABA_*A*_ receptor-mediated inhibition most likely contributes to forward suppression, while at longer ISIs other mechanisms such as synaptic depression appear to play a major role (Wehr and Zador, 2005). However, GABA_*B*_ receptors have been shown to similarly control paired-pulse suppression (Porter and Nieves, 2004). Earlier work suggested that not only inhibitory transmitter systems, but also metabotropic glutamate receptors are involved in regulating paired-pulse behavior (von Gersdorff et al., 1997). In addition, Zucker and Regehr (2002) had shown that vesicle depletion of calcium buffers and accompanying changes of release probabilities of excitatory synapses are implicated in paired-pulse behavior. Finally, the application of GABA_*A*_ agonists such as lorazepam in studies of human motor cortex (Ziemann et al., 1996; Werhahn et al., 1999) and somatosensory cortex (Huttunen et al., 2008; Stude et al., 2016) have provided rather direct evidence that GABAergic mechanisms are crucial for controlling paired-pulse behavior.

## Paired-pulse suppression as a cortical phenomenon

Paired-pulse behavior differs fundamentally between cortical and subcortical neurons, with little paired-pulse suppression found subcortically. It has therefore been argued that the profound cortical paired-pulse suppression is unlikely a consequence of the temporal thalamic response properties (Wehr and Zador, 2005). Comparing the temporal response properties along the sensory pathway of the auditory system supported the view that paired-pulse suppression is most likely generated at a cortical level (Creutzfeldt et al., 1980; Miller et al., 2002). Multichannel SEP-recordings made in human somatosensory system following paired median nerve stimulation showed that paired-pulse suppression is present at least upstream to the brainstem nuclei (Höffken et al., 2010, 2013a). Together these findings support the view that the mechanisms mediating paired-pulse suppression operate most likely in primary cortices and beyond.

## Other markers of excitation-inhibition

There are many markers used in human research that are utilized to obtain information about the role and amount of inhibitory processes. Magnetic resonance spectroscopy (MRS) is a relatively novel technique that allows measuring GABA levels reliably in humans *in vivo* (Puts and Edden, 2012). MRS is a non-invasive neuroimaging technique to detect the concentration of metabolites in a specific brain region, including glutamate, glutamine, GABA, and others (Puts and Edden, 2012). Employing this method, over the last years, fascinating insight has been obtained in the involvement of GABAergic mechanisms in learning, cognition, behavior and disease (Li et al., 2022). A particular advantage of the *in vivo* nature of this technique is to study GABA concentrations during development and across the lifespan (Saleh et al., 2020; Porges et al., 2021).

In a recent study utilizing MRS glutamate and GABA concentrations were measured to obtain insight into possible differences of visual perceptual training-induced learning and perceptual changes induced by high-frequency rTMS application. It was found that the time courses of the changes of GABA and glutamate differed as well as the contribution of both transmitters suggesting different underlying mechanisms, although both approaches involve changes in excitation and inhibition (Lin et al., 2023).

When transcranial magnetic stimulation is applied over the occipital lobe, it evokes brief light sensations, so-called phosphenes. Therefore, thresholds of phosphene induction can be measured and expressed as relative stimulator outputs, which has been used as a simple, non-invasive method to assess excitability in human visual cortex (Kammer, 1999; Sparing et al., 2005). Measuring phosphene thresholds by means of TMS has been employed in many studies addressing altered excitability in human visual cortex. For example, enhanced excitability levels were reported in patients suffering from migraine (Afra et al., 1998; Aurora et al., 2003; Gerwig et al., 2005), after medical treatment with anticonvulsants (Artemenko et al., 2008; Palermo et al., 2009) and light deprivation (Boroojerdi et al., 2000; Pitskel et al., 2007). Interestingly, there is a significant correlation between the individual phosphene threshold and paired-pulse suppression recorded in central VI indicating a significant link between both measures (Höffken et al., 2013b).

**FIGURE 2:**
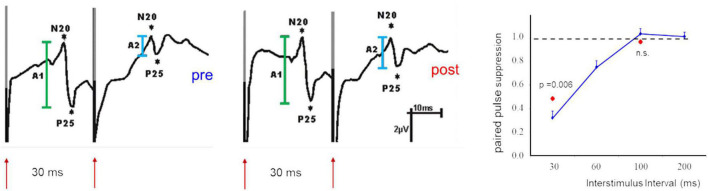
Example of paired-pulse suppression recorded after paired-median nerve stimulation in SI before (pre, **left**) and after (post, **middle**) application of a facilitatory stimulation protocol. Shown are SEP components of the N20//P25 complex (first response in green, second response in light blue). Note that the response amplitudes of the first stimulus are not affected. The recovery curve **(right)** illustrates the dependence of paired-pulse suppression from the ISI used. The second response remains also unaffected when long ISIs (100 ms) are used. Changes of paired-pulse suppression are limited to short ISIs and are exclusively due to changes of the second response amplitude [modified from Höffken et al. (2007)].

In addition, the absolute amplitudes of VEPs have been used as an indicator of changes of cortical excitability (Kaneda et al., 1997; Yilmaz et al., 1998; Bohotin et al., 2002; Avitabile et al., 2007). Besides the difficulties in obtaining reproducible response amplitudes in VEPs there is a major difference between EPs recorded following a single stimulus and the paired-pulse suppression. The suppression ratio between the first and the second response is a relative measure and thus more independent against noise. Most importantly, changes of paired pulse suppression are due to alterations of the suppression of the second response with no or little changes of the response amplitude of the first (cf. Figure 2). This can be interpreted that changes of the suppression reflects intracortical processing, while changes of the first response amplitude rather reflect changes of the afferent input.

Alpha oscillations in the range of 10 Hz typically found in visual or somatosensory cortex play a critical role in gating information processing by suppressing irrelevant information (Klimesch et al., 2007; Jensen and Mazaheri, 2010; Haegens et al., 2011a,b; Jensen et al., 2014; Zrenner et al., 2018). Alpha oscillations can be recorded either in occipital areas referred to as visual alpha, or in somatosensory cortex, referred to as mu-rhythm. Independent of location, higher alpha power is associated with better performance, excitability or evoked activity suggesting that the increased alpha activity is a signature of top down inhibitory control known as “gating by inhibition” (Pfurtscheller and Lopes da Silva, 1999; Klimesch et al., 2007; Jensen and Mazaheri, 2010).

For example, involvement in a difficult working memory task has been demonstrated to be associated with higher alpha power levels in cortical regions not engaged in the task (Klimesch et al., 1999). Similarly, somatosensory alpha power fluctuations on a trial by trial bases have been observed in association with the allocation of neural resources. As a result, alpha power could be shown to influences and to predict tactile task performance (Linkenkaer-Hansen et al., 2004). Pre-stimulus alpha oscillations of low to intermediate power that were recorded in cortical areas engaged in behavioral tasks were shown to enhance performance (Ai and Roy, 2014; Baumgarten et al., 2016). Furthermore, during a visual training task, not only pre-stimulus alpha but also alpha desynchronization increased significantly with training (Bays et al., 2015). These findings support the view that the regulation of pre-stimulus alpha levels serves as a recruiting mechanism optimizing efficient processing.

Animal models allow for a more invasive way of analysis of inhibitory processes such as recording of membrane potential changes, or assessment by immunohistochemical or chromatographic methods. A particular phenomenon reflecting intracellular inhibition was revealed by real-time optical imaging using voltage-sensitive dyes (Sharon and Grinvald, 2002). When presenting visual stimuli with different orientation a small transient drop in the rate at which the evoked response increased coincided with maximal selectivity to the stimulus. This deceleration and subsequent acceleration termed evoked deceleration-acceleration (DA) notch has been interpreted as a signature of a temporary suppression (Sharon and Grinvald, 2002; Kozyrev et al., 2014).

While all these markers reflect inhibitory processes, their relationship is far from being clear, and substantial research is required to unravel their potential interrelationship.

## Bidirectional changes of paired-pulse suppression parallel changes of perceptual performance

Insight into the role of inhibitory processes and on the emergence of plasticity and learning requires simultaneous assessment of inhibition-excitation markers and of perceptual performance. Transcranial magnetic stimulation (TMS) approaches were first used in studies exploring effects of TMS on cortical excitability (Kujirai et al., 1993) and motor learning. It was found that muscle training of the flexor policis brevis improved force and acceleration of movement, which was associated with an increase in motor evoked potential (MEP) amplitudes (Liepert et al., 1998; Muellbacher et al., 2001). Most notable, using low-frequency repetitive TMS (rTMS) reduced motor cortex excitability (Muellbacher et al., 2000). These early studies showed for the first time a link between excitability and performance, as well as between different TMS frequencies and excitability. Recent studies using entorhinohippocampal slice cultures showed that application of 10-Hz rTMS reduced GABAergic synaptic strength (Lenz et al., 2016). For a review of potential cellular mechanisms see Siebner et al. (2022), and Bolognini et al. (2009), Fricke et al. (2011), Polanía et al. (2018), Antal et al. (2022) for reviews about motor cortex plasticity and methods of non-invasive brain stimulation.

## Somatosensory cortex and tactile perception

Studies exploring plastic changes of somatosensory cortical excitability and tactile perception have similarly employed low- or high frequency rTMS. Application of high-frequency 10 Hz rTMS to the finger representation of primary SI improved tactile spatial discrimination performance of the index finger that outlasted the stimulation period for several hours (Tegenthoff et al., 2005). These data were the first demonstrating an improvement in human sensory performance by direct stimulation of the brain from outside. The changes in performance were accompanied by an enlargement of the cortical map representing the index finger. Moreover, the changes in the cortical map correlated with the individually observed gain in performance induced by rTMS. Separate experiments using paired-pulse stimulation techniques in combination with recording SEPs had shown that application of 5 Hz rTMS reduced paired-pulse suppression indicative for enhanced excitability in SI (Ragert et al., 2004; Gatica Tossi et al., 2013a,b). Comparable findings were observed after application of intermittent theta burst rTMS over human primary somatosensory cortex, where tactile discrimination improved parallel to enhanced cortical excitability (Ragert et al., 2008a).

In contrast, low frequency ∼1 Hz rTMS applied over SI finger representation had been found to impair tactile perception of the hand (Knecht et al., 2003; Satow et al., 2003; Tegenthoff et al., 2006). The impairment of tactile spatial discrimination performance outlasted the period of stimulation for a few hours and was accompanied by an enhancement of paired-pulse suppression indicating reduced excitability in SI (Tegenthoff et al., 2006). These data showed that application of rTMS at low or high frequencies induce lasting bidirectional changes in excitability and in tactile perceptual performance.

In daily live, human learning is driven by practicing and repetition. In contrast, in laboratory *in vitro* studies learning is induced merely by electrical stimulation in the absence of attention and motivation. Under these conditions, timing and temporal structure of pulse trains play a crucial role resulting in the induction of long-term potentiation and long-term depression - LTP and LTD (Bliss and Collingridge, 1993; Lynch, 2004; Malenka and Bear, 2004). To close the gap between these extremes a approach has been developed that translates protocols that very effectively evoke plastic changes *in vitro* into sensory stimulation protocols that can be applied in human participants. These so-called long-term potentiation-like or long-term depression-like sensory stimulation protocol alter persistently human perceptual performance in parallel to changes of neural processing. Most importantly, these changes are evoked without explicit task training and without attention (see review: Dinse and Tegenthoff, 2019). Recordings made in the hand representation of SI during the 30 min of application showed that each train of stimuli evokes a transient series of SEPs which after about 500 ms reach a 20 Hz steady-state response. This response pattern is maintained during the entire 2-s-train, with no evoked activity during the inter-train period. Most notably, there is no evidence for a response habituation over the 40 min of stimulation (Brickwedde et al., 2020). When this type of repetitive sensory stimulation is applied to the fingers of a hand, the tactile acuity, i.e., the spatial tactile discrimination performance of the stimulated finger is improved (Godde et al., 2000) as well as haptic performance (Dinse et al., 2005). The neural substrates underlying these perceptual changes include map reorganization in the hand representation in primary and secondary somatosensory cortex (Pleger et al., 2001, 2003; Dinse et al., 2003), changes of cortical excitability (Höffken et al., 2007), and changes in gray matter volume (Schmidt-Wilcke et al., 2018). In all cases, neural changes correlated with the amount of improvement in tactile acuity, such as increased BOLD (blood oxygenation level dependent) signals or cortical map changes implicating a strong link between perceptual changes and those observed a neural level (Pleger et al., 2001, 2003; Dinse et al., 2003; Höffken et al., 2007; Schmidt-Wilcke et al., 2018).

In contrast to the application of 30 min of a high-frequent intermittent finger stimulation protocol, which improved tactile acuity (Ragert et al., 2008b), the application of a low-frequency (1 Hz) protocol impaired tactile discrimination (Ragert et al., 2008b). Simultaneous assessment of paired-pulse suppression in SI revealed an overall reduction of paired-pulse suppression. This increase in excitability correlated positively with the individual gain in performance, indicating higher excitability in good learners (Höffken et al., 2007). The opposite observation was made when a low-frequency stimulation protocol was used. In this case, paired-pulse suppression was increased parallel to impaired tactile discrimination (Gatica Tossi and Dinse, 2008). The general property of changes of paired-pulse suppression following a facilitatory stimulation protocol are illustrated in Figure 2.

**FIGURE 3:**
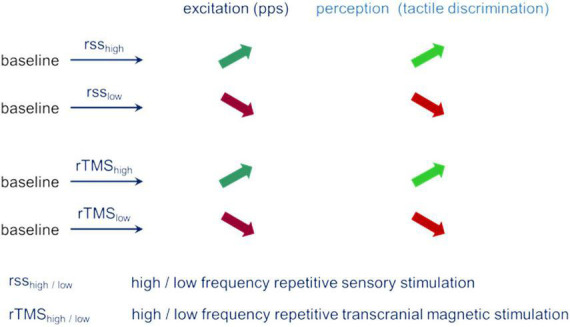
Parallel changes of excitation (pps – paired-pulse suppression) and perception (tactile spatial discrimination) for different stimulation conditions.

The similar pattern of parallel changes of performance and excitability observed independent of the type of evoking plastic changes provides strong evidence for a fundamental role excitability appears to play during plastic changes and changes in perceptual performance (Figure 3).

This view is further supported by a study that combined the application of high-frequency rTMS with application of repetitive tactile stimulation (Ragert et al., 2003). Both types of interventions are known to increase excitability, therefore, if high levels of excitability are linked to high perceptual gain, an even stronger gain in perceptual improvement could be expected. This is exactly what happened: Combined application boosted the gain in improvement (22% vs. 15%). Most notable, this boost in performance after combined application depended on the effectiveness of the repetitive tactile stimulation protocol when applied alone. Participants, who improved only little in tactile performance after the single application, showed the largest improvement after combined application. This observation suggests that the effects seen after combined application were specific for poor learners (Ragert et al., 2003).

## Visual cortex and visual perception

Compared to SI and the sense of touch, less human data are available about the combined assessment of changes of visual cortical excitability and visual perceptual performance. However, by putting together the findings from different studies related to plasticity, perceptual learning and excitability changes a very similar pattern emerges as described for the sense of touch and somatosensory plasticity processes.

While there are abundant reports about visual perceptual learning, few addressed the link to excitability changes (Seitz and Dinse, 2007; Sagi, 2010; Sasaki et al., 2010). Neary et al. (2005) showed that training of oriented lines over several days improved discrimination performance. This typical perceptual learning effect was accompanied by a reduction of the amount of TMS suppression indicative of reduced inhibition, or enhanced excitation in visual cortex. Other studies showed that using non-invasive brain stimulation protocols improve visual perceptual performance without concomitant visual input in a way similar to that described for somatosensory system (Tegenthoff et al., 2005). For example, applying 10 Hz rTMS over visual cortex improved visual orientation discrimination (Klaes et al., 2003). A similar finding was obtained when anodal transcranial direct current stimulation (tDCS) was applied over 4 consecutive days over visual cortex (Sczesny-Kaiser et al., 2016). Orientation discrimination improved, but not for kathodal tDCS. Most importantly, the discrimination improvement was paralleled by a decrease in paired-pulse suppression of VEPs and of phosphene thresholds, both supporting that the visual learning was associated with an increase of visual cortical excitability.

Repetitive sensory stimulation was also used for studies of the plasticity of the visual system. Here, instead of repetitive stimulating the index finger, visual patterned stimulation was applied (Marzoll et al., 2018, 2022). When for 40 min a low-frequency 1 Hz stimulation was applied, visual orientation discrimination was impaired, while a 20 Hz, intermittent stimulation improved orientation discrimination (Marzoll et al., 2018). These bidirectional changes in performance resemble those observed in somatosensory cortex (Ragert et al., 2008b). While for the somatosensory system, parallel changes of de- and increased excitability had been reported, comparable studies are missing so far for the visual system.

Brain stimulation techniques have also been used in animal studies addressing plastic changes of orientation preference maps (OPMs) in primary visual cortex and potential mechanisms underlying these plastic changes. Intracortical high-frequency micro-stimulation has been shown to reliably drive plastic changes of receptive fields and of cortical maps within a few hours (Nudo et al., 1990). Applying this technique in visual cortex demonstrated that the layout of OPM could be selectively altered (Godde et al., 2002). Further insight into the plastic processes induced by high-frequency stimulation was provided by a study combining real-time optical imaging and TMS. It was found that single pulse TMS large portion of cortical regions were strongly suppressed, while 10 Hz rTMS lead to enhanced spontaneous activity accompanied by wide-spread reduction of inhibition. Importantly, subsequent visual stimulation showed signs of long-term potentiation (Kozyrev et al., 2014). Furthermore, this state of enhanced excitability appears to facilitate plastic reorganization. When a given orientation was presented during this state, OPMs were altered in way that the normally balanced representation of all orientations were shifted toward the stimulated orientation (Kozyrev et al., 2018).

Taken together, the available data from the visual system demonstrate that different forms of inhibition-excitation markers are altered during the development of plastic processes consistent with the view that improved perceptual abilities emerge during states of enhanced excitability. The apparent similarity to the observations made in the somatosensory system makes it conceivable that these processes therefore might reflect a general property of sensory plastic processes underlying perceptual learning.

## Excitability changes during aging

During aging, brains change in numerous ways. A prominent characteristic is the overall decline in inhibitory mechanisms that go in parallel to a deterioration of perceptual and cognitive performance (Schmolesky et al., 2000; Poe et al., 2001; Leventhal et al., 2003; Hua et al., 2006). In fact, paired-pulse suppression in SI has been found to be reduced in aged rats (David-Jürgens and Dinse, 2010), and motor cortex in elderly healthy participants (Peinemann et al., 2001; Oliviero et al., 2006; Smith et al., 2009). A study in SI in elderly participants combining measurement of paired-pulse suppression with assessment of tactile acuity demonstrated that age-related reduction of paired-pulse suppression was correlated with the age-related decline in spatial discrimination performance (Lenz et al., 2012). These data showed that age-related enhanced excitability was associated with poor perceptual performance, seemingly contradicting the observations summarized above for young adults.

To reconcile these findings made in young adult and elderly participants computer simulations using a mean-field model of cortical activation (Wilson and Cowan, 1973) was used (Pleger et al., 2016). In this model, cortical population activity depends on two factors, the distance between inputs, here the skin, and Mexican-hat-type interactions mediated by local excitation and broad inhibition (Wilson and Cowan, 1973). The presence of either uni- or bimodal field-responses was regarded as an equivalent of the “one” or “two” response perceptual decisions in the psychophysical assessment. Learning and plasticity processes as seen in young adults were modeled by a decreased amount of inhibitory interaction. In contrast, effects of aging were simulated by a broadening of the inhibitory interaction. In the simulations, both “young” and “old” models give rise to a net increase in excitability in line with the empirical data, but the resulting performance differs: While in the young model the interaction leads to focused, spatially circumscribed activations allowing fine spatial discrimination, the old model results in broad, smeared distributions of activation hindering discrimination. According to these simulations, the structure of intracortical excitatory and inhibitory interaction is differentially affected by aging and learning processes explaining the size of cortical representation, the amount of cortical excitability, as well as the outcome of perceptual tasks.

## Inhibition markers as predictors of subsequent performance changes

It is common wisdom that there are good and poor learners, but the reasons that individuals are characterized by a tremendous learning variability remains to a large extent unclear (Fahle and Henke-Fahle, 1996). In addition to attention and motivation (Dayan and Balleine, 2002; Ahissar and Hochstein, 2004), other features have been found that predict large fractions of learning variability such as genetic polymorphisms (Kleim et al., 2006; Cheeran et al., 2008) or cortical gray matter thickness (Conde et al., 2012).

**FIGURE 4:**
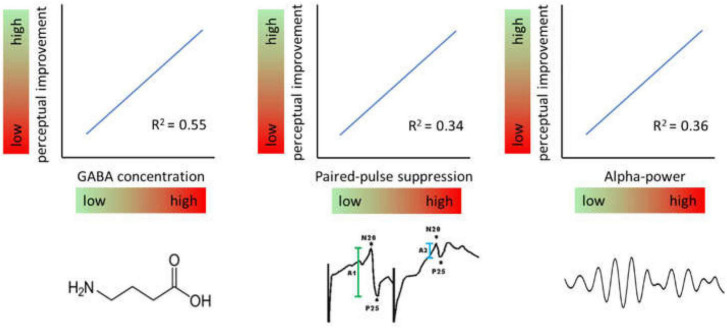
Effects of baseline levels of different markers of inhibition. Generally, high levels of inhibition measured immediately prior to induction of a learning protocol predict high levels of perceptual improvement and vice versa.

Recent studies have shown that also the excitatory state prior to learning can predict a substantial amount of variability. According to these studies, high levels of baseline inhibition as measured immediately before the induction of perceptual learning is associated with largest perceptual improvements (Figure 4). This property ascribes another important function to excitation-inhibition processes in plasticity and perceptual learning.

In a study measuring the time course of GABA concentrations by MRS in a voxel located in hand representation of SI, participants with the highest GABA concentrations showed largest gain in learning outcome (Heba et al., 2016). By that about 50% of the entire learning variance was explained. A related observation had been made in respect to baseline somatosensory alpha oscillations which have been implicated in gating information processing by suppressing irrelevant information. Recordings of ongoing EEG over the hand representation in SI showed that maximal improvement in a subsequent learning tasks was correlated with individual highest alpha power predicting 35% of the learning outcome (Freyer et al., 2013). Interestingly, the analysis of other frequency bands such as the beta and lower gamma band revealed no comparable dependencies suggesting a special role for alpha frequencies. More recently it was shown that paired-pulse suppression in SI is a similar predictor. Low excitability, i.e., high paired-pulse suppression was associated with high learning outcome explaining around 30% of the variability (Höffken et al., in preparation).

These findings raise two interesting questions. First, the fact that the inhibition markers described explain more than 100% of the total learning variability suggest that they might to some extent be interrelated and rely on rather similar or even identical cellular mechanisms. For example it is presently not clear in how far paired-pulse suppression is linked to GABA concentrations or alpha oscillations. Interestingly, a recent study combined measuring paired-pulse suppression in SI with measuring GABA levels using magnetic resonance spectroscopy (Cheng et al., 2017). They found no correlation between suppression and GABA concentrations, which might indicate that a possible link between both might be more complex. However, due to methodological differences in paired-pulse assessment (using of P35 SEP components, ISI of 500 ms) these data are difficult to reconcile with the technical approach followed in the Höffken et al. (2008) studies.

Second, if it is true that a given inhibition marker predicts up to 50% of the learning variance, and if there is a causal relationship, it is conceivable that manipulating this marker should result in even better learning outcome. This hypothesis had been tested in respect to somatosensory alpha oscillations.

**FIGURE 5:**
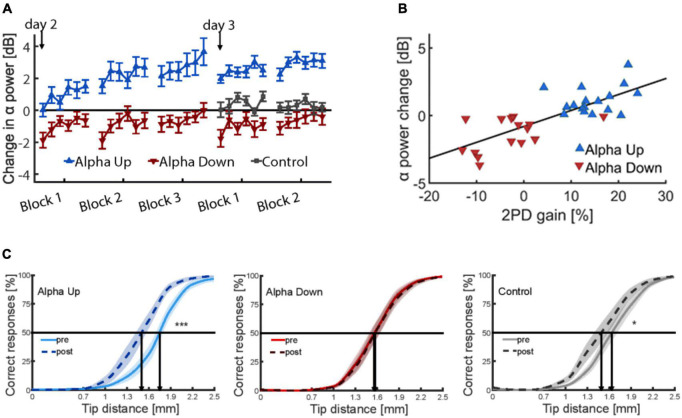
**(A)** Time course of alpha power changes in somatosensory cortex (mu rhythm) on both training days, relative to the first baseline measure on day one (0 dB), revealing considerable training effects of alpha. **(B)** Correlation between neurofeedback-induced changes of alpha-power and percent changes in discrimination performance explaining 59% of the variance. **(C)** Psychometric curves depicting tactile acuity before and after 20 min of repetitive sensory stimulation. Learning effects are indicated by a shift of the curve to the left. Data are presented as mean ± SEM. *p < 0.05; ***p < 0.001. The alpha-up group shows significant enhancement of improvement compared to controls, while participants of the alpha-down group show no improvement (Brickwedde et al., 2019).

Brain oscillations such as alpha are subject to targeted modification through neurofeedback techniques (Gruzelier et al., 2006; Salari et al., 2012). In the past, neurofeedback (NF) training has been employed to improve cognitive abilities and working and episodic memory performance (Hanslmayr et al., 2005; Zoefel et al., 2011; Hsueh et al., 2016). Also, NF training is used in the treatment of ADHD and epileptic seizures (Sterman, 1981; Egner and Sterman, 2006). Therefore, to make an argument that tactile perceptual learning is indeed caused by changes in individual alpha power, a newly developed neurofeedback protocol was used to up- or down regulate somatosensory alpha within a few sessions (Brickwedde et al., 2019). In brief, these data showed that the largest learning outcome was observed in individuals with highest increase in alpha power (Figure 5).

In contrast, participants who were trained in the alpha down group to decrease their alpha power, were not able to improve discrimination performance implying that learning processes had been blocked in this group. The results of a control group were in between. As a main result, baseline alpha power before learning predicted 59% of the interindividual learning variability (Brickwedde et al., 2019). In contrast, under spontaneous conditions, alpha power explained only 35% of the variance (Freyer et al., 2013) indicating a substantial advantage through the NF training.

## Conclusion

The balance of inhibition and excitation plays a fundamental role in stabilizing brain processing and is critically involved in plasticity and learning. We here addressed the role of excitatory states and inhibition in somatosensory and visual cortex during various forms of perceptual learning. Generally, studies in human participants requires indirect and non-invasive approaches to assess states of excitation and inhibition. We summarized different approaches used such as the assessment of paired-pulse behavior, GABA concentrations by MRS, phosphene induction by TMS or the measurement of alpha oscillations. We emphasize that the interrelatedness of the different excitation-inhibition markers is currently poorly understood requiring substantial research. Simultaneous recordings of paired-pulse behavior in SI and Vi imply that cortical excitability characterizes the currently prevailing excitatory state within a circumscribed patch of cortex. We reviewed the available evidence for parallel and simultaneous changes of excitability and perceptual performance following various approaches that can alter perception in a specific way. We demonstrated that in both SI and VI improved perceptual abilities are accompanied by enhanced excitation, while impaired perception is associated with enhanced inhibition. In addition, recent findings demonstrated that the baseline states of excitation and inhibition measured immediately before a perceptual learning protocol can predict the learning outcome to a considerable degree. This bears important implications for targeted interventions aiming at enhancing learning outcome by altering baseline states of excitation. We summarize first attempts that successfully employed neurofeedback training to up- and down-regulate alpha power prior to learning, which resulted in a significant enhancement of learning. High learning efficacy is an essential requirement for successful clinical rehabilitation measures and in school education. Accordingly, neurofeedback training or other interventions targeting baseline states of excitation could be prime candidates to enhance learning capabilities not only under laboratory conditions but also in everyday life situations.

## Author contributions

HD wrote the first draft of the manuscript. All authors performed the conceptualization, methods, data collection and analysis, commented on previous versions of the manuscript, contributed to the study conception and design, and read and approved the final manuscript.

## References

[B1] Abbott L. Nelson S. (2000). Synaptic plasticity: Taming the beast. *Nat. Neurosci.* 3 1178–1183. 10.1038/81453 11127835

[B2] Afra J. Mascia A. Gerard P. Maertens de Noordhout A. Schoenen J. (1998). Interictal cortical excitability in migraine: A study using transcranial magnetic stimulation of motor and visual cortices. *Ann. Neurol.* 44 209–215. 10.1002/ana.410440211 9708543

[B3] Ahissar M. Hochstein S. (2004). The reverse hierarchy theory of visual perceptual learning. *Trends Cogn. Sci.* 8 457–464. 10.1016/j.tics.2004.08.011 15450510

[B4] Ai L. Roy T. (2014). The phase of prestimulus alpha oscillations affects tactile perception. *J. Neurosci.* 111 1300–1307. 10.1152/jn.00125.2013 24381028

[B5] Allison T. (1962). Recovery functions of somatosensory evoked responses in man. *Electroencephalogr. Clin. Neurophysiol.* 14 331–343. 10.1016/0013-4694(62)90110-4 13860632

[B6] Antal A. Luber B. Brem A. Bikson M. Brunoni A. Cohen Kadosh R. (2022). Non-invasive brain stimulation and neuroenhancement. *Clin. Neurophysiol. Pract.* 7 146–165. 10.1016/j.cnp.2022.05.002 35734582 PMC9207555

[B7] Artemenko A. Kurenkov A. Filatova E. Nikitin S. Kaube H. Katsarava Z. (2008). Effects of topiramate on migraine frequency and cortical excitability in patients with frequent migraine. *Cephalalgia* 28 203–208. 10.1111/j.1468-2982.2007.01491.x 18254890

[B8] Aurora S. Welch K. Al-Sayed F. (2003). The threshold for phosphenes is lower in migraine. *Cephalalgia* 23 258–263. 10.1046/j.1468-2982.2003.00471.x 12716342

[B9] Avitabile T. Longo A. Caruso S. Gagliano C. Amato R. Scollo D. (2007). Changes in visual evoked potentials during the menstrual cycle in young women. *Curr. Eye Res.* 32 999–1003. 10.1080/02713680701679006 18027176

[B10] Barron H. (2021). Neural inhibition for continual learning and memory. *Curr. Opin. Neurobiol.* 67 85–94. 10.1016/j.conb.2020.09.007 33129012 PMC7116367

[B11] Baumgarten T. Schnitzler A. Lange J. (2016). Prestimulus alpha power influences tactile temporal perceptual discrimination and confidence in decisions. *Cereb. Cortex* 26 891–903. 10.1093/cercor/bhu247 25331603

[B12] Bays B. Visscher K. Le Dantec C. Seitz A. (2015). Alpha-band EEG activity in perceptual learning. *J. Vis.* 15:7. 10.1167/15.10.7 26370167 PMC4570730

[B13] Bliss T. Collingridge G. (1993). A synaptic model of memory: Long-term potentiation in the hippocampus. *Nature* 361 31–39. 10.1038/361031a0 8421494

[B14] Bohotin V. Fumal A. Vandenheede M. Gérard P. Bohotin C. Maertens de Noordhout A. (2002). Effects of repetitive transcranial magnetic stimulation on visual evoked potentials in migraine. *Brain* 125 912–922. 10.1093/brain/awf081 11912123

[B15] Bolognini N. Pascual-Leone A. Fregni F. (2009). Using non-invasive brain stimulation to augment motor training-induced plasticity. *J. Neuroeng. Rehabil.* 6:8. 10.1186/1743-0003-6-8 19292910 PMC2667408

[B16] Boroojerdi B. Bushara K. Corwell B. Immisch I. Battaglia F. Muellbacher W. (2000). Enhanced excitability of the human visual cortex induced by short-term light deprivation. *Cereb. Cortex* 10 529–534. 10.1093/cercor/10.5.529 10847602

[B17] Brickwedde M. Krüger M. Dinse H. (2019). Somatosensory alpha oscillations gate perceptual learning efficiency. *Nat. Commun.* 10:263. 10.1038/s41467-018-08012-0 30651567 PMC6335466

[B18] Brickwedde M. Schmidt M. Krüger M. Dinse H. (2020). 20 Hz steady-state response in somatosensory cortex during induction of tactile perceptual learning through LTP-like sensory stimulation. *Front. Hum. Neurosci.* 14:257. 10.3389/fnhum.2020.00257 32694988 PMC7339616

[B19] Carcea I. Froemke R. (2013). Cortical plasticity, excitatory-inhibitory balance, and sensory perception. *Prog. Brain Res.* 207 65–90. 10.1016/B978-0-444-63327-9.00003-5 24309251 PMC4300113

[B20] Cheeran B. Talelli P. Mori F. Koch G. Suppa A. Edwards M. (2008). A common polymorphism in the brain-derived neurotrophic factor gene (BDNF) modulates human cortical plasticity and the response to rTMS. *J. Physiol.* 23 5717–5725. 10.1113/jphysiol.2008.159905 18845611 PMC2655403

[B21] Cheng C. H. Tsai S. Y. Liu C. Y. Niddam D. M. (2017). Automatic inhibitory function in the human somatosensory and motor cortices: An MEG-MRS study. *Sci. Rep*. 7:4234. 10.1038/s41598-017-04564-1 PMC5484662 28652623

[B22] Conde V. Vollmann H. Sehm B. Taubert M. Villringer A. Ragert P. (2012). Cortical thickness in primary sensorimotor cortex influences the effectiveness of paired associative stimulation. *Neuroimage* 60 864–870. 10.1016/j.neuroimage.2012.01.052 22266412

[B23] Creutzfeldt O. Hellweg F. Schreiner C. (1980). Thalamocortical transformation of responses to complex auditory stimuli. *Exp. Brain Res.* 39 87–104. 10.1007/bf00237072 6247179

[B24] David-Jürgens M. Dinse H. (2010). Effects of aging on paired-pulse behavior of rat somatosensory cortical neurons. *Cereb. Cortex* 20 1208–1216. 10.1093/cercor/bhp185 19745019 PMC2852507

[B25] Dayan P. Balleine B. (2002). Reward, motivation, and reinforcement learning. *Neuron* 2 285–298. 10.1016/S0896-6273(02)00963-7 12383782

[B26] Dinse H. Kalisch T. Ragert P. Pleger B. Schwenkreis P. Tegenthoff M. (2005). Improving human haptic performance in normal and impaired human populations through unattended activation-based learning. *Trans. Appl. Perc.* 2 71–88. 10.1145/1060581.1060583

[B27] Dinse H. Ragert P. Pleger B. Schwenkreis P. Tegenthoff M. (2003). Pharmacological modulation of perceptual learning and associated cortical reorganization. *Science* 301 91–94. 10.1126/science.1085423 12843392

[B28] Dinse H. R. Tegenthoff M. (2019). “Repetitive sensory stimulation – A canonical approach to control the induction of human learning at a behavioural and neural level,” in *Handbook of in vivo neural plasticity techniques. A systems neuroscience approach to the neural basis of memory and cognition*, Vol. 28, ed. Manahan-Vaughan D. (Cambridge: Academic Press), 389–413.

[B29] Egner T. Sterman M. (2006). Neurofeedback treatment of epilepsy: From basic rationale to practical application. *Expert Rev. Neurother.* 2 247–257. 10.1586/14737175.6.2.247 16466304

[B30] Fahle M. Henke-Fahle S. (1996). Interobserver variance in perceptual performance and learning. *Invest Ophthalmol. Vis. Sci.* 37 869–877. 8603871

[B31] Feldman D. (2009). Synaptic mechanisms for plasticity in neocortex. *Annu. Rev. Neurosci.* 32 33–55. 10.1146/annurev.neuro.051508.135516 19400721 PMC3071739

[B32] Freyer F. Becker R. Dinse H. Ritter P. (2013). State-dependent perceptual learning. *J. Neurosci.* 33 2900–2907. 10.1523/JNEUROSCI.4039-12.2013 23407948 PMC6619196

[B33] Fricke K. Seeber A. Thirugnanasambandam N. Paulus W. Nitsche M. Rothwell J. (2011). Time course of the induction of homeostatic plasticity generated by repeated transcranial direct current stimulation of the human motor cortex. *J. Neurophysiol.* 1053 1141–1149. 10.1152/jn.00608.2009 21177994

[B34] Gatica Tossi M. Dinse H. (2008). Biderectional plastic changes of tactile perception and somatosensory cortex excitability induced by peripheral short-interval paired stimulation. *Soc. Neurosci. Abstr.* 177:1.

[B35] Gatica Tossi M. Lillemeier A. Dinse H. (2013a). Influence of stimulation intensity on paired-pulse suppression of human median nerve somatosensory evoked potentials. *Neuroreport* 24 451–456. 10.1097/WNR.0b013e3283616378 23660631

[B36] Gatica Tossi M. Stude P. Schwenkreis P. Tegenthoff M. Dinse H. (2013b). Behavioural and neurophysiological markers reveal differential sensitivity to homeostatic interactions between centrally and peripherally applied passive stimulation. *Eur. J. Neurosci.* 38 2893–2901. 10.1111/ejn.12293 23834757

[B37] Gerwig M. Niehaus L. Kastrup O. Stude P. Diener H. (2005). Visual cortex excitability in migraine evaluated by single and paired magnetic stimuli. *Headache* 45 1394–1399. 10.1111/j.1526-4610.2005.00272.x 16324172

[B38] Godde B. Leonhardt R. Cords S. Dinse H. (2002). Plasticity of orientation preference maps in the visual cortex of adult cats. *Proc. Natl. Acad. Sci. U.S.A.* 99 6352–6357. 10.1073/pnas.082407499 11959906 PMC122952

[B39] Godde B. Stauffenberg B. Spengler F. Dinse H. (2000). Tactile coactivation induced changes in spatial discrimination performance. *J. Neurosci.* 20 1597–1604. 10662849 10.1523/JNEUROSCI.20-04-01597.2000 PMC6772356

[B40] Gruzelier J. Egner T. Vernon D. (2006). Validating the efficacy of neurofeedback for optimising performance. *Prog. Brain Res.* 159 421–431. 10.1016/S0079-6123(06)59027-2 17071246

[B41] Haegens S. Handel B. Jensen O. (2011a). Top-down controlled alpha band activity in somatosensory areas determines behavioral performance in a discrimination task. *J. Neurosci.* 31 5197–5204. 10.1523/JNEUROSCI.5199-10.2011 21471354 PMC6622699

[B42] Haegens S. Nácher V. Luna R. Romo R. Jensen O. (2011b). α-Oscillations in the monkey sensorimotor network influence discrimination performance by rhythmical inhibition of neuronal spiking. *Proc. Natl. Acad. Sci. U.S.A.* 108 19377–19382. 10.1073/pnas.1117190108 22084106 PMC3228466

[B43] Hanslmayr S. Sauseng P. Doppelmayr M. Schabus M. Klimesch W. (2005). Increasing individual upper alpha power by neurofeedback improves cognitive performance in human subjects. *Appl. Psychophysiol. Biofeedback* 30 1–10. 10.1007/s10484-005-2169-8 15889581

[B44] Heba S. Puts N. Kalisch T. Glaubitz B. Haag L. Lenz M. (2016). Local GABA concentration predicts perceptual improvements after repetitive sensory stimulation in humans. *Cereb. Cortex* 26 1295–1301. 26637451 10.1093/cercor/bhv296 PMC4737612

[B45] Höffken O. Grehl T. Dinse H. Tegenthoff M. Bach M. (2008). Paired-pulse behavior of visually evoked potentials recorded in human visual cortex using patterned paired-pulse stimulation. *Exp. Brain Res.* 188 427–435. 10.1007/s00221-008-1374-0 18427792

[B46] Höffken O. Lenz M. Tegenthoff M. Schwenkreis P. (2010). Multichannel SEP-recording after paired median nerve stimulation suggests origin of paired-pulse inhibition rostral of the brainstem. *Neurosci. Lett.* 468 308–311. 10.1016/j.neulet.2009.11.021 19914346

[B47] Höffken O. Stude P. Lenz M. Bach M. Dinse H. Tegenthoff M. (2009). Visual paired-pulse stimulation reveals enhanced visual cortex excitability in migraineurs. *Eur. J. Neurosci.* 30 714–720. 10.1111/j.1460-9568.2009.06859.x 19674086

[B48] Höffken O. Tannwitz J. Lenz M. Sczesny-Kaiser M. Tegenthoff M. Schwenkreis P. (2013a). Influence of parameter settings on paired-pulse-suppression in somatosensory evoked potentials: A systematic analysis. *Clin. Neurophysiol.* 124 574–580. 10.1016/j.clinph.2012.08.012 22995592

[B49] Höffken O. Lenz M. Sczesny-Kaiser M. Dinse H. Tegenthoff M. (2013b). Phosphene thresholds correlate with paired-pulse suppression of visually evoked potentials. *Brain Stimul.* 6 118–121. 10.1016/j.brs.2012.02.004 22445534

[B50] Höffken O. Tegenthoff M. Dinse H. R. (in preparation). Baseline paired-pulse suppression predicts tactile perceptual learning.

[B51] Höffken O. Veit M. Knossalla F. Lissek S. Bliem B. Ragert P. (2007). Sustained increase of somatosensory cortex excitability by tactile coactivation studied by paired median nerve stimulation in humans correlates with perceptual gain. *J. Physiol.* 584 463–471. 10.1113/jphysiol.2007.140079 17702814 PMC2277142

[B52] Hsueh J. Chen T. Chen J. Shaw F. (2016). Neurofeedback training of EEG alpha rhythm enhances episodic and working memory. *Hum. Brain Mapp.* 37 2662–2675. 27038114 10.1002/hbm.23201 PMC6867560

[B53] Hua T. Li X. He L. Zhou Y. Wang Y. Leventhal A. (2006). Functional degradation of visual cortical cells in old cats. *Neurobiol. Aging* 27 155–162. 16298251 10.1016/j.neurobiolaging.2004.11.012

[B54] Huttunen J. Pekkonen E. Kivisaari R. Autti T. Kahkonen S. (2008). Modulation of somatosensory evoked fields from SI and SII by acute GABA A-agonism and paired-pulse stimulation. *Neuroimage* 40 427–434. 10.1016/j.neuroimage.2007.12.024 18234513

[B55] Jensen O. Mazaheri A. (2010). Shaping functional architecture by oscillatory alpha activity: Gating by inhibition. *Front. Hum. Neurosci.* 4:186. 10.3389/fnhum.2010.00186 21119777 PMC2990626

[B56] Jensen O. Gips B. Bergmann T. Bonnefond M. (2014). Temporal coding organized by coupled alpha and gamma oscillations prioritize visual processing. *Trends Neurosci.* 37 357–369. 24836381 10.1016/j.tins.2014.04.001

[B57] Kaas J. Merzenich M. Killackey H. (1983). The reorganization of somatosensory cortex following peripheral nerve damage in adult and developing mammals. *Annu. Rev. Neurosci.* 6 325–356. 10.1146/annurev.ne.06.030183.001545 6340591

[B58] Kammer T. (1999). Phosphenes and transient scotomas induced by magnetic stimulation of the occipital lobe: Their topographic relationship. *Neuropsychologia* 37 191–198. 10.1016/s0028-3932(98)00093-1 10080376

[B59] Kaneda Y. Ikuta T. Nakayama H. Kagawa K. Furuta N. (1997). Visual evoked potential and electroencephalogram of healthy females during the menstrual cycle. *J. Med. Invest.* 44 41–46. 9395716

[B60] Klaes C. Ragert P. Jancke D. Tegenthoff M. Dinse H. (2003). rTMS induced improvement of human orientation discrimination. *Soc. Neurosci. Abstr.* 911:22.

[B61] Kleim J. Chan S. Pringle E. Schallert K. Procaccio V. Jimenez R. (2006). BDNF val66met polymorphism is associated with modified experience-dependent plasticity in human motor cortex. *Nat. Neurosci.* 9 735–357. 10.1038/nn1699 16680163

[B62] Klimesch W. Doppelmayr M. Schwaiger J. Auinger P. Winkler T. (1999). ‘Paradoxical‘ alpha synchronization in a memory task. *Brain Res. Cogn. Brain Res.* 7 93–501. 10.1016/s0926-6410(98)00056-1 10076094

[B63] Klimesch W. Sauseng P. Hanslmayr S. (2007). EEG alpha oscillations: The inhibition-timing hypothesis. *Brain Res. Rev.* 53 63–88. 16887192 10.1016/j.brainresrev.2006.06.003

[B64] Knecht S. Ellger T. Breitenstein C. Bernd Ringelstein E. Henningsen H. (2003). Changing cortical excitability with low-frequency transcranial magnetic stimulation can induce sustained disruption of tactile perception. *Biol. Psychiatry* 53 175–179. 10.1016/s0006322302013823 12547474

[B65] Kozyrev V. Eysel U. Jancke D. (2014). Voltage-sensitive dye imaging of transcranial magnetic stimulation-induced intracortical dynamics. *Proc Natl Acad Sci U S A* 111 13553–13558. 10.1073/pnas.1405508111 25187557 PMC4169942

[B66] Kozyrev V. Staadt R. Eysel U. Jancke D. (2018). TMS-induced neuronal plasticity enables targeted remodeling of visual cortical maps. *Proc. Natl. Acad. Sci. U.S.A.* 115 6476–6481. 10.1073/pnas.1802798115 29866856 PMC6016806

[B67] Kujirai T. Caramia M. Rothwell J. Day B. Thompson P. Ferbert A. (1993). Corticocortical inhibition in human motor cortex. *J. Physiol.* 471 501–519. 8120818 10.1113/jphysiol.1993.sp019912 PMC1143973

[B68] Lenz M. Galanis C. Müller-Dahlhaus F. Opitz A. Wierenga C. Szabó G. (2016). Repetitive magnetic stimulation induces plasticity of inhibitory synapses. *Nat. Commun.* 7:10020. 10.1038/ncomms10020 PMC4729863 26743822

[B69] Lenz M. Tegenthoff M. Kohlhaas K. Stude P. Höffken O. Gatica Tossi M. (2012). Increased excitability of somatosensory cortex in aged humans is associated with impaired tactile acuity. *J. Neurosci.* 32 1811–1816. 10.1523/JNEUROSCI.2722-11.2012 22302820 PMC6703354

[B70] Leventhal A. Wang Y. Pu M. Zhou Y. Ma Y. (2003). GABA and its agonists improved visual cortical function in senescent monkeys. *Science* 300 812–815. 12730605 10.1126/science.1082874

[B71] Li H. Heise K. Chalavi S. Puts N. Edden R. Swinnen S. (2022). The role of MRS-assessed GABA in human behavioral performance. *Prog. Neurobiol.* 212:102247. 10.1016/j.pneurobio.2022.102247 35149113

[B72] Liepert J. Classen J. Cohen L. Hallett M. (1998). Task-dependent changes of intracortical inhibition. *Exp. Brain Res.* 118 421–426. 10.1007/s002210050296 9497149

[B73] Lin S. Lien Y. Shibata K. Sasaki Y. Watanabe T. Lin C. (2023). The phase of plasticity-induced neurochemical changes of high-frequency repetitive transcranial magnetic stimulation are different from visual perceptual learning. *Sci. Rep.* 13:5720. 10.1038/s41598-023-32985-8 37029245 PMC10082079

[B74] Linkenkaer-Hansen K. Nikulin V. Palva S. Ilmoniemi R. Palva J. (2004). Prestimulus oscillations enhance psychophysical performance in humans. *J. Neurosci.* 24 10186–10190. 15537890 10.1523/JNEUROSCI.2584-04.2004 PMC6730198

[B75] Lynch M. (2004). Long-term potentiation and memory. *Physiol. Rev.* 84 87–136. 14715912 10.1152/physrev.00014.2003

[B76] Malenka R. Bear M. (2004). LTP and LTD: An embarrassment of riches. *Neuron* 44 5–21. 15450156 10.1016/j.neuron.2004.09.012

[B77] Marzoll A. Saygi T. Dinse H. (2018). The effect of LTP- and LTD-like visual stimulation on modulation of human orientation discrimination. *Sci. Rep.* 8:16156. 10.1038/s41598-018-34276-z 30385849 PMC6212525

[B78] Marzoll A. Shibata K. Toyoizumi T. Chavva I. Watanabe T. (2022). Decrease in signal-related activity by visual training and repetitive visual stimulation. *iScience* 25:105492. 10.1016/j.isci.2022.105492 36419854 PMC9676190

[B79] Miller L. Escabi M. Read H. Schreiner C. (2002). Spectrotemporal receptive fields in the lemniscal auditory thalamus and cortex. *J. Neurophysiol.* 87 516–527. 10.1152/jn.00395.2001 11784767

[B80] Muellbacher W. Ziemann U. Boroojerdi B. Hallett M. (2000). Effects of low-frequency transcranial magnetic stimulation on motor excitability and basic motor behavior. *Clin. Neurophysiol.* 111 1002–1007. 10.1016/s1388-2457(00)00284-4 10825706

[B81] Muellbacher W. Ziemann U. Boroojerdi B. Cohen L. Hallett M. (2001). Role of the human motor cortex in rapid motor learning. *Exp. Brain Res.* 136 431–438. 10.1007/s002210000614 11291723

[B82] Neary K. Anand S. Hotson J. (2005). Perceptual learning of line orientation modifies the effects of transcranial magnetic stimulation of visual cortex. *Exp. Brain Res.* 162 23–34. 10.1007/s00221-004-2117-5 15578168

[B83] Nudo R. Jenkins W. Merzenich M. (1990). Repetitive microstimulation alters the cortical representation of movements in adult rats. *Somatosens. Mot. Res.* 7 463–483. 10.3109/08990229009144720 2291378

[B84] Oliviero A. Profice P. Tonali P. Pilato F. Saturno E. Dileone M. (2006). Effects of aging on motor cortex excitability. *Neurosci. Res.* 55 74–77. 16584795 10.1016/j.neures.2006.02.002

[B85] Palermo A. Fierro B. Giglia G. Cosentino G. Puma A. Brighina F. (2009). Modulation of visual cortex excitability in migraine with aura: Effects of valproate therapy. *Neurosci. Lett.* 467 26–29. 10.1016/j.neulet.2009.09.054 19800389

[B86] Peinemann A. Lehner C. Conrad B. Siebner H. (2001). Age-related decrease in paired-pulse intracortical inhibition in the human primary motor cortex. *Neurosci. Lett.* 313 33–36. 11684333 10.1016/s0304-3940(01)02239-x

[B87] Pfurtscheller G. Lopes da Silva F. (1999). Event-related EEG/MEG synchronization and desynchronization: Basic principles. *Clin. Neurophysiol.* 110 1842–1857. 10.1016/s1388-2457(99)00141-8 10576479

[B88] Pitskel N. Merabet L. Ramos-Estebanez C. Kauffman T. Pascual-Leone A. (2007). Time-dependent changes in cortical excitability after prolonged visual deprivation. *Neuroreport* 18 1703–1707. 10.1097/WNR.0b013e3282f0d2c1 17921872

[B89] Pleger B. Dinse H. Ragert P. Schwenkreis P. Malin J. Tegenthoff M. (2001). Shifts in cortical representations predict human discrimination improvement. *Proc. Natl. Acad. Soc. U.S.A.* 98 12255–12260. 10.1073/pnas.191176298 PMC59801 11593042

[B90] Pleger B. Foerster A. Ragert P. Dinse H. Schwenkreis P. Malin J. (2003). Functional imaging of perceptual learning in human primary and secondary somatosensory cortex. *Neuron* 40 643–653. 14642286 10.1016/s0896-6273(03)00677-9

[B91] Pleger B. Wilimzig C. Nicolas V. Kalisch T. Ragert P. Tegenthoff M. (2016). A complementary role of intracortical inhibition in age-related tactile degradation and its remodelling in humans. *Sci. Rep.* 6:27388. 10.1038/srep27388 27302219 PMC4908433

[B92] Poe B. Linville C. Brunso-Bechtold J. (2001). Age-related decline of presumptive inhibitory synapses in the sensorimotor cortex as revealed by the physical disector. *J. Comp. Neurol.* 439 65–72. 11579382 10.1002/cne.1335

[B93] Polanía R. Nitsche M. Ruff C. (2018). Studying and modifying brain function with non-invasive brain stimulation. *Nat. Neurosci.* 21 174–187. 10.1038/s41593-017-0054-4 29311747

[B94] Porges E. Jensen G. Foster B. Edden R. Puts N. (2021). The trajectory of cortical GABA across the lifespan, an individual participant data meta-analysis of edited MRS studies. *Elife* 10:e62575. 10.7554/eLife.62575 34061022 PMC8225386

[B95] Porter J. Nieves D. (2004). Presynaptic GABAB receptors modulate thalamic excitation of inhibitory and excitatory neurons in the mouse barrel cortex. *J. Neurophysiol.* 92 2762–2770. 10.1152/jn.00196.2004 15254073 PMC3677950

[B96] Puts N. Edden R. (2012). In vivo magnetic resonance spectroscopy of GABA: A methodological review. *Prog. Nucl. Magn. Reson. Spectrosc.* 60 29–41. 10.1016/j.pnmrs.2011.06.001 22293397 PMC3383792

[B97] Ragert P. Becker M. Tegenthoff M. Pleger B. Dinse H. (2004). Sustained increase of somatosensory cortex excitability by 5 Hz repetitive transcranial magnetic stimulation studied by paired median nerve stimulation in humans. *Neurosci. Lett.* 356 91–94. 10.1016/j.neulet.2003.11.034 14746871

[B98] Ragert P. Dinse H. Pleger B. Wilimzig C. Frombach E. Schwenkreis P. (2003). Combination of 5 Hz repetitive transcranial magnetic stimulation (rTMS) and tactile coactivation boosts tactile discrimination in humans. *Neurosci. Lett.* 348 105–108. 10.1016/s0304-3940(03)00745-6 12902029

[B99] Ragert P. Franzkowiak S. Schwenkreis P. Tegenthoff M. Dinse H. (2008a). Improvement of tactile perception and enhancement of cortical excitability through intermittent theta burst rTMS over human primary somatosensory cortex. *Exp. Brain Res.* 184 1–11. 10.1007/s00221-007-1073-2 17680239

[B100] Ragert P. Kalisch T. Bliem B. Franzkowiak S. Dinse H. (2008b). Differential effects of tactile high- and low-frequency stimulation on tactile discrimination in human subjects. *BMC Neurosci.* 9:9. 10.1186/1471-2202-9-9 18215277 PMC2244613

[B101] Reis J. Swayne O. Vandermeeren Y. Camus M. Dimyan M. Harris-Love M. (2008). Contribution of transcranial magnetic stimulation to the understanding of cortical mechanisms involved in motor control. *J. Physiol.* 586 325–351. 10.1113/jphysiol.2007.144824 17974592 PMC2375593

[B102] Sagi D. (2010). Perceptual learning in vision research. *Vision Res.* 51 1552–1566. 10.1016/j.visres.2010.10.019 20974167

[B103] Salari N. Büchel C. Rose M. (2012). Functional dissociation of ongoing oscillatory brain states. *PLoS One* 5:e38090. 10.1371/journal.pone.0038090 22666454 PMC3364191

[B104] Saleh M. Papantoni A. Mikkelsen M. Hui S. Oeltzschner G. Puts N. (2020). Effect of age on GABA+ and glutathione in a pediatric sample. *Am. J. Neuroradiol.* 41 1099–1104. 10.3174/ajnr.A6543 32381543 PMC7342763

[B105] Sasaki Y. Nanez J. Watanabe T. (2010). Advances in visual perceptual learning and plasticity. *Nat. Rev. Neurosci.* 11 53–60. 10.1038/nrn2737 19953104 PMC2864603

[B106] Satow T. Mima T. Yamamoto J. Oga T. Begum T. Aso T. (2003). Short-lasting impairment of tactile perception by 0.9Hz-rTMS of the sensorimotor cortex. *Neurology* 60 1045–1047. 12654982 10.1212/01.wnl.0000052821.99580.d3

[B107] Schloemer N. Lenz M. Tegenthoff M. Dinse H. Höffken O. (2020). Parallel modulation of intracortical excitability of somatosensory and visual cortex by the gonadal hormones estradiol and progesterone. *Sci. Rep.* 10:22237. 10.1038/s41598-020-79389-6 33335211 PMC7747729

[B108] Schmidt-Wilcke T. Wulms N. Heba S. Pleger B. Puts N. Glaubitz B. (2018). Structural changes in brain morphology induced by brief periods of repetitive sensory stimulation. *Neuroimage* 165 148–157. 10.1016/j.neuroimage.2017.10.016 29031533

[B109] Schmolesky M. Wang Y. Pu M. Leventhal A. (2000). Degradation of stimulus selectivity of visual cortical cells in senescent rhesus monkeys. *Nat. Neurosci.* 3 384–390. 10725929 10.1038/73957

[B110] Sczesny-Kaiser M. Beckhaus K. Dinse H. Schwenkreis P. Tegenthoff M. Höffken O. (2016). Repetitive transcranial direct current stimulation induced excitability changes of primary visual cortex and visual learning effects-A pilot study. *Front. Behav. Neurosci.* 10:116. 10.3389/fnbeh.2016.00116 27375452 PMC4891342

[B111] Seitz A. Dinse H. (2007). A common framework for perceptual learning. *Curr. Opin. Neurobiol.* 17 148–153. 10.1016/j.conb.2007.02.004 17317151

[B112] Sharon D. Grinvald A. (2002). Dynamics and constancy in cortical spatiotemporal patterns of orientation processing. *Science* 295 512–515. 10.1126/science.1065916 11799249

[B113] Siebner H. Funke K. Aberra A. Antal A. Bestmann S. Chen R. (2022). Transcranial magnetic stimulation of the brain: What is stimulated? - A consensus and critical position paper. *Clin. Neurophysiol.* 140 59–97. 10.1016/j.clinph.2022.04.022 35738037 PMC9753778

[B114] Smith A. Ridding M. Higgins R. Wittert G. Pitcher J. (2009). Age-related changes in short-latency motor cortex inhibition. *Exp. Brain Res.* 198 489–500. 19618169 10.1007/s00221-009-1945-8

[B115] Sparing R. Dambeck N. Stock K. Meister I. Huetter D. Boroojerdi B. (2005). Investigation of the primary visual cortex using short-interval paired-pulse transcranial magnetic stimulation (TMS). *Neurosci. Lett.* 382 312–316. 10.1016/j.neulet.2005.03.036 15925110

[B116] Sterman M. (1981). EEG biofeedback: Physiological behavior modification. *Neurosci. Biobehav. Rev.* 5 405–412. 7301228 10.1016/0149-7634(81)90036-1

[B117] Stude P. Lenz M. Höffken O. Tegenthoff M. Dinse H. (2016). A single dose of lorazepam reduces paired-pulse suppression of median nerve evoked somatosensory evoked potentials. *Eur. J. Neurosci.* 43 1156–1160. 10.1111/ejn.13224 26929110

[B118] Tegenthoff M. Franzkowiak S. Ragert P. Schwenkreis P. Dinse H. (2006). Differential effects of high and low-frequency rTMS on different tactile perceptual tasks and on cortical excitability of human somatosensory cortex (SI). *Soc. Neurosci. Abstr.* 32:53.28.

[B119] Tegenthoff M. Ragert P. Pleger B. Schwenkreis P. Förster A. Nicolas V. (2005). Improvement of tactile discrimination performance and enlargement of cortical somatosensory maps after 5 Hz rTMS. *PLoS Biol.* 3:e362. 10.1371/journal.pbio.0030362 16218766 PMC1255742

[B120] Turrigiano G. Nelson S. (2000). Hebb and homeostasis in neuronal plasticity. *Curr. Opin. Neurobiol.* 10 358–364. 10.1016/s0959-4388(00)00091-x 10851171

[B121] von Gersdorff H. Schneggenburger R. Weis S. Neher E. (1997). Presynaptic depression at a calyx synapse: The small contribution of metabotropic glutamate receptors. *J. Neurosci.* 17 8137–8146. 10.1523/JNEUROSCI.17-21-08137.1997 9334389 PMC6573755

[B122] Wehr M. Zador A. (2005). Synaptic mechanisms of forward suppression in rat auditory cortex. *Neuron* 47 437–445. 10.1016/j.neuron.2005.06.009 16055066

[B123] Werhahn K. Kunesch E. Noachtar S. Benecke R. Classen J. (1999). Differential effects on motorcortical inhibition induced by blockade of GABA uptake in humans. *J. Physiol.* 517 591–597. 10.1111/j.1469-7793.1999.0591t.x 10332104 PMC2269337

[B124] Wilson H. Cowan J. (1973). A mathematical theory of the functional dynamics of cortical and thalamic nervous tissue. *Kybernetik* 13 55–80. 4767470 10.1007/BF00288786

[B125] Yilmaz H. Erkin E. Mavioglu H. Sungurtekin U. (1998). Changes in pattern reversal evoked potentials during menstrual cycle. *Int. Ophthalmol.* 22 27–30. 10.1023/a:1006165126702 10090445

[B126] Ziemann U. Lonnecker S. Steinhoff B. Paulus W. (1996). The effect of lorazepam on the motor cortical excitability in man. *Exp. Brain Res.* 109 127–135. 10.1007/bf00228633 8740215

[B127] Zoefel B. Huster R. Herrmann C. S. (2011). Neurofeedback training of the upper alpha frequency band in EEG improves cognitive performance. *Neuroimage* 54 1427–1431. 20850552 10.1016/j.neuroimage.2010.08.078

[B128] Zrenner C. Desideri D. Belardinelli P. Ziemann U. (2018). Real-time EEG-defined excitability states determine efficacy of TMS-induced plasticity in human motor cortex. *Brain Stimul.* 11 374–389. 29191438 10.1016/j.brs.2017.11.016

[B129] Zucker R. Regehr W. G. (2002). Short-term synaptic plasticity. *Annu. Rev. Physiol.* 64 355–405. 10.1146/annurev.physiol.64.092501.114547 11826273

